# Modeling cartilage pathology in mucopolysaccharidosis VI using iPSCs reveals early dysregulation of chondrogenic and metabolic gene expression

**DOI:** 10.3389/fbioe.2022.949063

**Published:** 2022-12-06

**Authors:** M. Broeders, Jgj van Rooij, E. Oussoren, Tjm van Gestel, Ca Smith, Sj Kimber, Rm Verdijk, Maem Wagenmakers, Jmp van den Hout, At van der Ploeg, R. Narcisi, Wwmp Pijnappel

**Affiliations:** ^1^ Department of Pediatrics, Erasmus MC University Medical Center, Rotterdam, Netherlands; ^2^ Department of Clinical Genetics, Erasmus MC University Medical Center, Rotterdam, Netherlands; ^3^ Center for Lysosomal and Metabolic Diseases, Erasmus MC University Medical Center, Rotterdam, Netherlands; ^4^ Department of Internal Medicine, Erasmus MC Medical Center, Rotterdam, Netherlands; ^5^ Division of Cell Matrix Biology and Regenerative Medicine, School of Biological Sciences, Faculty of Biology Medicine and Health, University of Manchester, Manchester, United Kingdom; ^6^ Department of Pathology, Erasmus MC University Medical Center, Rotterdam, Netherlands; ^7^ Department of Orthopaedics and Sports Medicine, Erasmus MC University Medical Center, Rotterdam, Netherlands

**Keywords:** mucopolysaccharidosis type VI, lysosomal storage disease, disease modeling, induced pluripotent stem cells, cartilage

## Abstract

Mucopolysaccharidosis type VI (MPS VI) is a metabolic disorder caused by disease-associated variants in the Arylsulfatase B (*ARSB*) gene, resulting in ARSB enzyme deficiency, lysosomal glycosaminoglycan accumulation, and cartilage and bone pathology. The molecular response to MPS VI that results in cartilage pathology in human patients is largely unknown. Here, we generated a disease model to study the early stages of cartilage pathology in MPS VI. We generated iPSCs from four patients and isogenic controls by inserting the *ARSB* cDNA in the *AAVS1* safe harbor locus using CRISPR/Cas9. Using an optimized chondrogenic differentiation protocol, we found Periodic acid–Schiff positive inclusions in hiPSC-derived chondrogenic cells with MPS VI. Genome-wide mRNA expression analysis showed that hiPSC-derived chondrogenic cells with MPS VI downregulated expression of genes involved in TGF-β/BMP signalling, and upregulated expression of inhibitors of the Wnt/β-catenin signalling pathway. Expression of genes involved in apoptosis and growth was upregulated, while expression of genes involved in glycosaminoglycan metabolism was dysregulated in hiPSC-derived chondrogenic cells with MPS VI. These results suggest that human *ARSB* deficiency in MPS VI causes changes in the transcriptional program underlying the early stages of chondrogenic differentiation and metabolism.

## 1 Introduction

Mucopolysaccharidoses type VI (MPS VI) is an autosomal recessive disorder caused by Arylsulfatase B (ARSB) enzyme deficiency leading to intralysosomal accumulation of the glycosaminoglycans (GAGs) dermatan sulfate (DS) and chondroitin sulfate (CS). MPS VI is a multisystemic disease with GAG accumulation in connective tissues and organs which ultimately leads to a cascade of symptoms such as corneal clouding, hepatosplenomegaly and bone and cartilage pathology. In all MPS VI patients the hips are frequently and severely affected resulting in limitations in mobility and pain with impact on quality of life (Oussoren et al., 2017).

Currently, treatment of MPS VI consists of enzyme replacement therapy (ERT) with intravenous administration of recombinant ARSB enzyme. Although ERT attenuates disease progression and resolves hepatosplenomegaly, the therapeutic effect on bone and cartilage pathology is limited. Cartilage in particular is nonresponsive to ERT, likely due to the poor vascularization of cartilage resulting in impaired delivery of the enzyme from the circulation to chondrocytes. In a pre-clinical setting, monthly intra-articular injection of ERT reduced storage material in articular cartilage of MPS VI cats (Auclair et al., 2006; Auclair et al., 2007). However, it is not feasible to inject individual joints monthly in every patient. Therefore, the cartilage pathology in MPS VI and other forms of MPS remains an unmet medical need to date.

The development of cartilage pathology in patients with MPS VI remains poorly understood. Several studies in animal models for MPS VI and related types of MPS have been performed, and have provided insight in the cascade of events resulting in cartilage pathology. This has shown that chrondrocytes in MPS VI are abnormal with a swollen appearance and containing membrane-bound inclusions/vacuoles (Haskins et al., 1980). In the growth plate, there is loss of columnar structure and excess of calcified cartilage (Abreu et al., 1995). In newborn mice abnormal anlagen of tracheal and articular cartilage was reported, indicating that cartilage pathology can already start during development (Evers et al., 1996). Studies from human cartilage biopsies of MPS I, II and III patients showed severe cartilage damage and abnormal chondrocytes (Oussoren et al., 2011; Oussoren et al., 2021), but limited information is available on the development of cartilage pathology in human patients with MPS VI.

At the molecular level, primary lysosomal accumulation of GAGs can result in secondary accumulation of cholesterol and the gangliosides GM2 and GM3, indicating cross talk between metabolic pathways in MPS and other lysosomal diseases (Ballabio and Gieselmann, 2009). GAGs can also function as receptor ligands to activate Toll like receptors, resulting in inflammation. In addition GAGs can also activate growth factors such as BMPs, involved in cartilage and bone development (Oussoren et al., 2011). In mice with the related disorder MPS VII, the growth plate was enlarged, disorganized, and contained fewer chondrocytes. It was suggested that chondrocytes displayed a delayed exit from G1 into M and a delayed terminal differentiation, possibly mediated by elevated expression of PTHrP and Wnt5a (Jiang et al., 2020). How these processes operate in human chondrocytes with MPS VI remains largely unknown.

In humans, the high variability of genetic background between individuals complicates the downstream analysis of disease progression (Soldner et al., 2011; Hockemeyer and Jaenisch, 2016). It has become clear that differences in gene expression and functional parameters can be considerable between individuals, which introduces a large amount of (seemingly random) variation to the analyses. Recent advancements in gene editing using CRISPR/Cas9 enable the generation of isogenic controls to reduce these effects and to decipher molecular mechanisms of disease by generating isogenic controls, i.e. diseased and healthy versions with the same genetic background (Broeders et al., 2019; Ernst et al., 2020). In contrast to mesenchymal stem cells (Kolf et al., 2007), hiPSCs have high capacity for self-renewal and are suitable for gene editing, which makes hiPSCs attractive for disease modeling of cartilage pathology.

Here, we generated isogenic pairs from four patient-derived hiPSC lines using CRISPR-Cas9 to generate a model for the cartilage pathology in MPS VI. We improved the protocol for chondrogenic differentiation of hiPSCs, and applied this to characterize the pathology and molecular changes in human chondrogenic cells with MPS VI.

## 2 Materials and methods

### 2.1 Ethics approval and consent to participate

The Institutional Review Board approved the study protocol, and all patients provided written informed consent.

### 2.2 LV-OSKM lentivirus production

HEK293T cells were cultured in 10 cm culture dishes with DMEM high glucose (Gibco) supplemented with 10% fetal bovine serum (Thermo Scientific) and 100 U/ml Penicillin/Streptomycin/Glutamine (Gibco). At 80% confluency, cells were transfected with 3 µg LV-OSKM reprogramming vector, 2 µg psPAX2 and 1 µg pVSV using Fugene 6 transfection reagent according to manufacturer’s protocol (Promega). Medium was filtered with 0.45 µm PDFV filters (Millipore) 72 h post transfection and concentrated by centrifugation for 2 h at 20 k rpm with a Beckman Coulter Ultracentrifuge with SW32 Ti rotor. The virus was dissolved in 100 µL concentration in DMEM low glucose (Gibco) and stored at −80°C.

### 2.3 hiPSC generation and culturing

HiPSCs were generated from patient-derived primary fibroblasts as described before (van der Wal et al., 2018). Fibroblast cells from four patients with a rapid disease progression were reprogrammed using a polycistronic lentiviral vector of Oct4, Sox2, Klf4, and c-Myc (LV-OSKM). hiPSCs were cultured on γ-irradiated mouse embryonic feeder (MEF) cells with hiPSC culture medium consisting of DMEM/F12 medium (Invitrogen), 20% knock-out serum replacement (Invitrogen), 1% non-essential amino acids (Gibco), 1% penicillin/streptomycin/L-glutamine (100x, Gibco), 2 mM β-mercaptoethanol (Invitrogen) and 20 ng/ml basic fibroblast growth factor 2 (Peprotech). After gene correction, the hiPSCs were transferred to a feeder free culture with Vitronectin XF (Stem Cell) as coating and mTeSR^™^ Plus (Stem Cell) as media. Healthy control hiPSCs were obtained from the HIPSCI database and cultured with Vitronectin XF (Stem Cell) as coating and mTeSR^™^ Plus (Stem Cell) as media. *Mycoplasma* tests were routinely performed on all cell lines using the MycoAlert^™^ *Mycoplasma* Detection Kit (Lonza) and were found negative.

### 2.4 Gene correction

The donor construct to express the healthy copy of the ARSB gene was generated as described previously (van der Wal et al., 2018; in ’t Groen et al., 2021), with the *ARSB* cDNA (from Sino biological, HG13674-G) instead of the *GAA* cDNA. CRISPR/Cas9 was used to insert the transgene into the AAVS1 locus. Gene editing was performed as described previously (van der Wal et al., 2018; in ’t Groen et al., 2021). In short, optimal target sites for the AAVS1 locus were selected using the CRISPRscan program (Moreno-Mateos et al., 2015). The 5′-GTC​ACC​AAT​CCT​GTC​CCT​AG-3′ sgRNA was selected and *in silico* analysis showed no potential off-targets with less than 4 mismatches (Supplementary Table S1). A vector containing the U6 promoter (addgene: 41,824) was used to express the sgRNA. hiPSCs on feeders were pretreated with RevitaCell (Thermo Fisher Scientific) 4 h prior nucleofection. Single cells were generated from hiPSC colonies by incubating with TrypLE (Thermo Fisher Scientific). A DNA mix was prepared consisting of 8.9 µg of pCAG-hCAS9-GFP (addgene: 44,719), 6.7 µg of TOPO-sgRNA, and 4.4 µg of donor vector. 2 × 10^6^ cells were nucleofected with the DNA mix using Amaxa Human Stem Cell Nucleofector Kit2 (VPH-5022, Lonza) with program B-016. Cells were plated in hiPSC-conditioned medium (hiPSC medium incubated for 24 h on feeder cells) supplemented with 20 ng/ml FGF2 (Prepotech) and RevitaCell to improve survival. 100 mg/mL G-418 (Invivogen) selection was initiated 48 h after nucleofection. Approximately 14 days after initiation of selection, single iPS colonies were picked and genotyped. Primers used for the cloning of the ARSB cDNA and genotyping of gene edited hiPSCs can be found in Supplementary Table S2.

### 2.5 Chondrogenic differentiation

HiPSCs were differentiated into chondrogenic cells using a 14-day differentiation protocol that was modified from Oldershaw et al. (Oldershaw et al., 2010). Single cells were generated from hiPSC colonies by incubation with TrypLE (Thermo Fisher Scientific), and 5 × 10^5^ cells were plated on a Vitronectin XF (Stem cell) coated plate. This protocol consists of activation of the WNT signaling using 2 µM CHIR99021 (R & D Systems) and 50 to 10 ng/ml Activin-A (Peprotech) from day 1–3.40 ng/ml FGF2 (Peprotech) was added from day 2 and sustained during the whole differentiation. 5 ng/ml BMP2 (R&D Systems) was added from day 3–10.1 µM SB431542 (Torcis) was sustained from day 5–8. GDF5 was added in increasing concentrations from day 9 onwards: 20 ng/ml on day 9 and 10, 40 ng/ml from day 11 until day 14. Cells were split on day 5 in a 1/5 to 1/8 ratio and on day 8 in a 1/4 to 1/6 ratio. Medium was supplemented with Revitacell on the day of splitting. Cell culture plates were coated with Vitronectin XF on days 1–7 and from day 8–14 with a Vitronectin XF and 0.1% gelatin mix (Sigma-Aldrich).

### 2.6 Periodic acid–Schiff staining

Day 14 chondrogenic cells were replated in 4 independent cultures on Cell culture plates that were coated with Vitronectin XF and 0.1% gelatin. After 48 h, cells were harvested by incubating with TrypLE (Thermo Fisher Scientific) and single cells were centrifuged on a slide using the Cytospin. The samples were fixed in a formaldehyde ethanol solution for 5 min and rinsed with water. Samples were then incubated in 1% Periodic acid (Supelco) for 10 min and rinsed with water for 10 min. The samples were placed in Schiffs reagent (Sigma-Aldrich) for 15 min at 37°C and rinsed with water for 15 min. The samples were then placed in Mayer’s hemalum solution (Sigma-Aldrich) for 5 min and rinsed with water. They were air-dried and mounted with Entellan (Sigma-Aldrich). Images were obtained using a NanoZoomer 2.0HT C9600-12 (Hamamatsu) and quantified by two independent observers.

### 2.7 ARSB enzyme assay

The ARSB enzyme activity was measured as described previously (Baum et al., 1959). In short, cells were lysed in TNE-1% (50 mM Tris-HCl pH 8.0, 150 mM NaCl, 50 mM NaF, and 1% Triton X100, containing a protease inhibitor cocktail (Roche) and added to the paranitrocatecholdisulfate substrate. The conversion of paranitrocatecholdisulfate to paranitrocatechol was measured on a spectrophotometer. A primary human skin fibroblast cell line from a healthy control was used as a reference.

### 2.8 RNA isolation and sequencing

RNA was isolated using the RNAeasy miniprep kit with on-column DNase treatment (Qiagen) according to manufacturer’s protocol. Sequencing was performed after poly-A selection and TruSeq library prep at the Human Genomics facility (www.glimdna.org) on a HiSeq2000 at paired-end 150 bp. Data were processed per sample using STAR (v2.3.0) (Dobin et al., 2013; Bolger et al., 2014), picard (v1.90), and GATK (v2.8). Transcript quantification was performed using featureCounts (v1.4.3) against all 57,820 gene features in GENCODE (version date; 2013-12-05) (Harrow et al., 2012; Liao et al., 2014).

### 2.9 Data analysis

Raw counts per gene were normalized using the edgeR (v3.8.6) trimmed mean of M-values method to counts per million values. Principal components (PCs) were calculated using “prcomp” in R, and then plotted to visually identify sample outliers. Statistical analysis was performed per gene using the glmFit function in edgeR, correcting stratifying for the four cell lines. Selected groups of genes are clustered and visualized using z-transformation per gene set and subsequent euclidean hierarchical clustering in the TIBCO Spotfire package (v7.14).

### 2.10 GO analysis

Differential expressed genes among isogenic controls were analyzed by the PANTHER Classification System (Mi et al., 2019). Selection for genes was based on a CPM of >0 for all samples and replicates, a log2 FC of 0.59 or -0.59 for upregulated and downregulated genes, respectively, and an FDR of ≤0.05. The flowchart for the selection of genes is shown in Supplementary Figure S4.

### 2.11 Western blot analysis

Total protein fractions were isolated using Mammalian Protein Extraction Reagent (M-PER) (#78501, Thermo Scientific) with 1X protease inhibitor (78,430, Thermo Scientific) and 1X phosphatase inhibitor (78,428, Thermo Scientific). Protein concentration was determined using the Pierce BCA Protein Assay Kit (23,225, Thermo Scientific). A total of 8 mg of total protein fraction for each sample was separated by a 4–15% precast polyacrylamide gel (5,671,084, Bio-Rad) and transferred to a nitrocellulose membrane (1,704,159, Bio-Rad) using the Trans-Blot Turbo Transfer System. Membranes were blocked in 5% dry milk powder in 0.1% tris-buffered saline-tween (TBS-T) for 2 h, washed three times in 0.1% TBS-T and incubated with primary antibodies against Cleaved Caspase-3, Non-phospho (Active) β-Catenin, α-Tubulin, or GAPDH (all diluted 1:1000; respectively 9,661, 8,814, 2,125 and 7,074, Cell Signaling Technology) overnight at 4°C following the manufacturer’s protocol. An anti-rabbit horseradish peroxidase (HPR)-linked secondary antibody (1:1000; 7,074, Cell Signaling Technology) was added and incubated for 1 h at room temperature. The blots were visualized with SuperSignal Chemiluminescent detection kit (34,077, Thermo Scientific) using the manufacturer’s instruction. Image analysis and quantification were performed using the National Institute of Health ImageJ freeware (release 1.53; http://rsb.info.nih.gov/ij/).

## 3 Results

### 3.1 Cartilage pathology in an MPS VI patient

**FIGURE 1:**
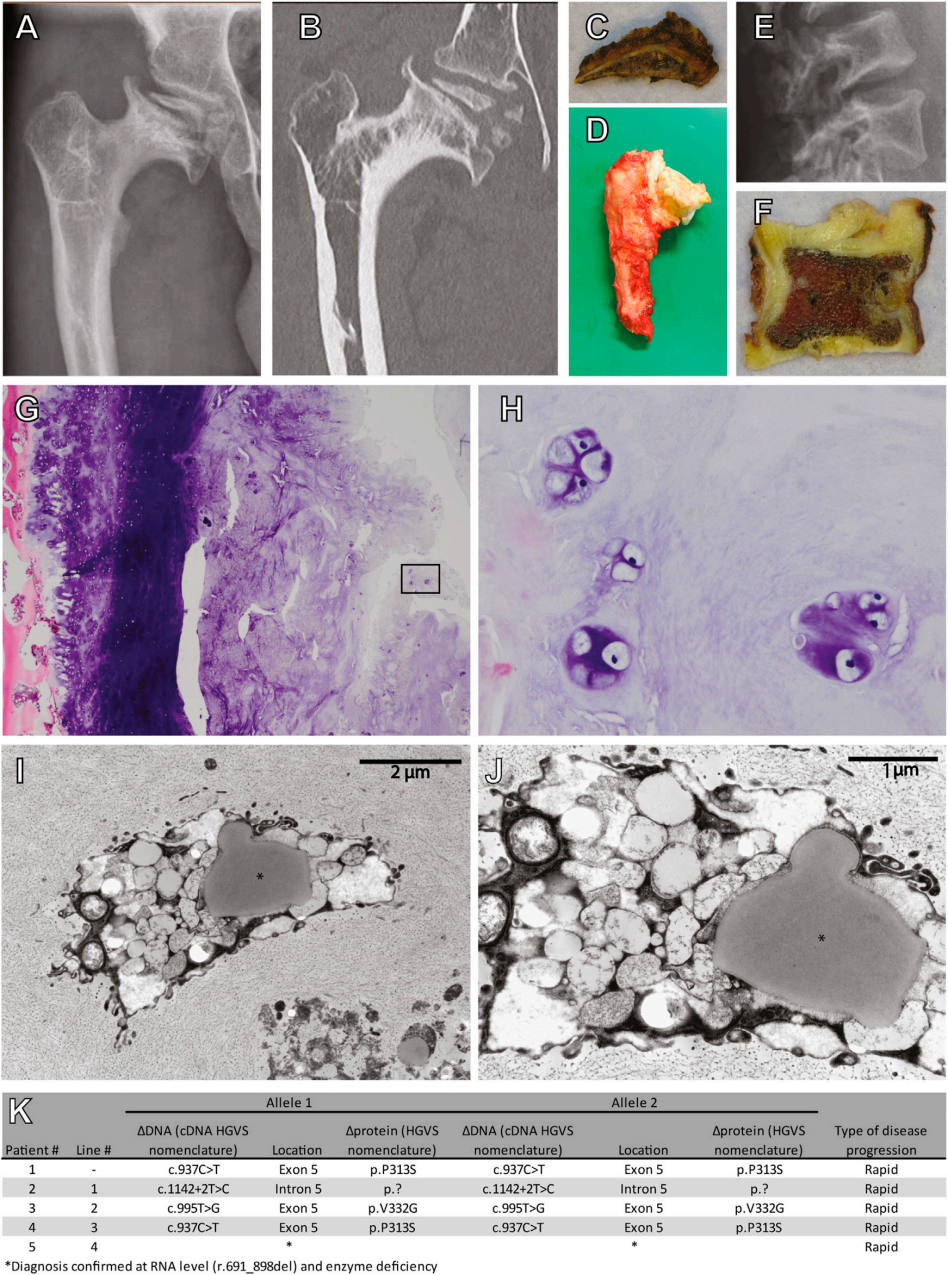
Cartilage pathology in a 25 year old diseased MPS VI patient. **(A)** X-ray and **(B)** CT scan of the right hip. Macroscopic pictures from autopsy of the **(C)** right acetabulum and **(D)** femoral head. **(E)** X-ray of the lumbar vertebrae. **(F)** A macroscopic picture from autopsy of one vertebra. **(G)** HE stain of cartilage from this vertebra (Magnification ×100), **(H)** details from the inset in G (Magnification ×400). **(I,J)** Electron microscopy analysis showing examples of chondroyctes with different types of vacuoles and in addition lipid storage (indicated with *). **(K)** Characteristics of included patients.

To study cartilage pathology in MPS VI, a femoral head and acetabulum were obtained by autopsy of a 25-year-old MPS VI patient that died from disease-related symptoms. This patient (Patient #1) had a homozygous *ARSB* c.937C>T disease-associated variant that lead to a rapid MPS VI disease progression. X-ray and CT analysis of the hip showed severe osteoarthritis of the femoral head with total destruction and abnormal ossification, and with an abnormal steep and dysplastic acetabulum, which is also shown in the macroscopic pictures (Figures 1A–D). The vertebrae were abnormal shaped, with an abnormal degenerative cartilage endplate (Figures 1E, F). Chondrocytes within the vertebrae cartilage showed multiple proliferative clones, which reflects the pathological response, and vacuolar changes in HE stained sections (Figures 1G,H). Electron microscopy analysis of the same cartilage showed examples of chondrocytes with different types of vacuoles and in addition lipid storage (Figures 1I,J). These results illustrate the joint pathology in MPS VI, and they show that chondrocytes are affected by very severe vacuolization and lipid storage.

## 3.2 Generation of patient-derived hiPSCs and gene editing to generate isogenic controls

To model cartilage pathology in human MPS VI, we selected four patients with a rapid disease progression. Diagnosis was based on ARSB enzyme activity, accumulation of urinary GAGs (uGAGs), the presence of disease-associated variants in the *ARSB* gene, and clinical symptoms (Figure 1K). Patient #2, and #3 were homozygous for *ARSB* c.1142+2T>C and c.995T>G, respectively. Patient #4 was homozygous for *ARSB* c.937C>T, and a sibling of patient #1. No disease-associated variant for patient #5 had been identified, but we recently confirmed the molecular diagnosis at the RNA level (Broeders et al., 2020). hiPSC lines were generated from primary fibroblasts using lentiviral expression of Oct4, Sox2, Klf-4, and C-Myc as described (van der Wal et al., 2018).

**FIGURE 2:**
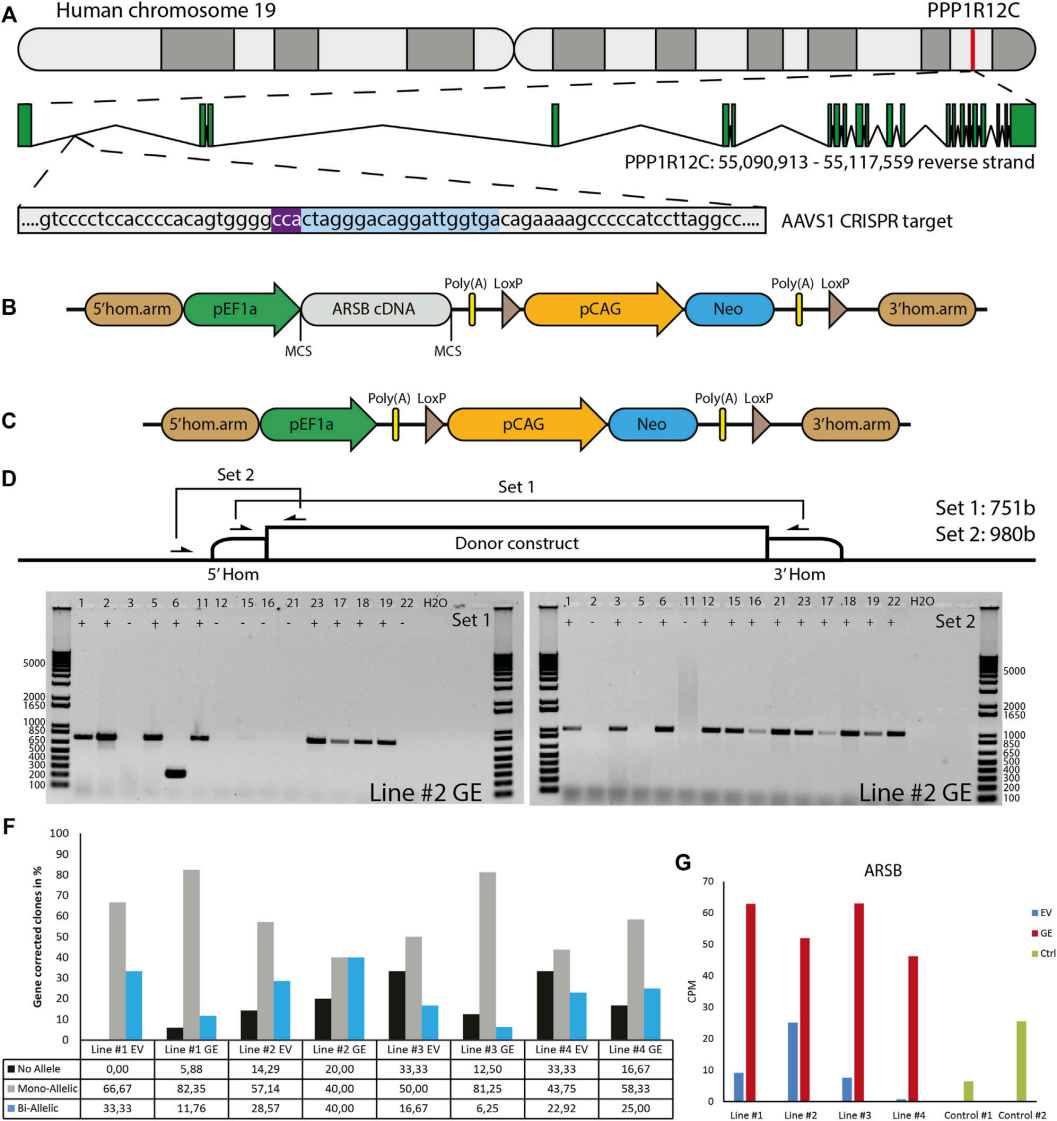
Gene editing in hiPSCs. **(A)** The gene editing strategy was designed to express *ARSB* from the AAVS1 locus. PAM sequence indicated in purple, gRNA target indicated in blue. **(B)** The donor construct generated for insertion of the ARSB cDNA in the AAVS1 safe harbor by CRISPR/Cas9-mediated gene editing. The neomycin cassette enables G418 selection of targeted colonies. **(C)** The empty vector construct used to generate isogenic controls. **(D)** Strategy for the PCR based genotyping of targeted colonies, primer set 1 spans the insertion site and only gives a product in the absence of targeting, primer set 2 amplifies a product only in the presence of the construct at the target site. **(E)** Typical genotyping result of picked colonies. With primer set 2, 12/15 colonies show successful mono-allelic or bi-allelic targeting. With primer set 1, 6/15 lack a PCR product, indicating a loss of the endogenous sequence. In combination colonies 3, 12, 15, 16, 21 and 22 show bi-allelic insertion of the construct. DNA Ladder: 1 Kb Plus DNA Ladder (Invitrogen) **(F)** Quantification of mono-allelic, bi-allelic and unsuccessfully targeted colonies. **(G)** *ARSB* mRNA expression in the selected bi-allelic targeted clones, determined using RNAseq. GE: Gene edited with ARSB cDNA, EV: Gene edited with empty vector control.

To restore expression of *ARSB*, a healthy copy was introduced into a safe location of the genome, also known as a safe harbor. This provides a generic and efficient method, which contrasts with correcting individual mutations. We chose the widely used *PPP1R12C* gene in the *AAVS1* locus located on chromosome 19 (Figure 2A). A donor construct previously described by us (van der Wal et al., 2018; in ’t Groen et al., 2021) was adapted to introduce a healthy copy of the *ARSB* gene using CRISPR/cas9 via the homology directed repair (HDR) pathway. This construct (Figure 2B) contained the *ARSB* cDNA driven by the constitutively active EF1α promoter, and the neomycin resistance gene to enable selection for successful integrations, which is required due to the highly inefficient HDR pathway. To validate *ARSB* expression by the donor construct, HeLa TK- cells were transfected and selection was started 24 h after transfection. After selection, the cells showed an ARSB enzyme activity of ∼1400 nmol/mg/hr, which was above the range seen in healthy control fibroblasts (Supplementary Figure S2). No ARSB enzyme activity was detected in HeLa TK- cells transfected with a pCAG-NEO control vector. This confirmed that the *ARSB* donor construct was functional.

To generate a control for the gene targeting procedure that did not change *ARSB* expression, we deleted the *ARSB* cDNA from the targeting vector (Figure 2C). hiPSCs were gene edited using either the *ARSB* cDNA vector (GE) or the empty targeting vector (EV) by co-transfection with an expression vector for human codon-optimized Cas9 nuclease, an expression vector for the guide RNA targeting the AAVS1 locus, and either targeting vector. After selection with G418, colonies were picked and genotyped using two PCR strategies; set 1 yields a product of 749 bp from the untargeted allele and 7,132 bp from the targeted allele. This 7,132 bp product is too large to be detected under the PCR conditions employed. Set 2 only yields a product of 980 bp if the *ARSB* cDNA is inserted in the targeted site (Figure 2D). A typical result is shown in Figure 2E, where the results of set 1 show that 8 out of 15 colonies still have the endogenous sequence at target site on one or both alleles. The results of set 2 show that 12 out of 15 colonies have the *ARSB* insertion at the targeted site. From these results we conclude that 6 colonies have both alleles targeted, 6 colonies have 1 allele targeted and 3 colonies have no insertion of the *ARSB* cDNA at the target site. The gene editing results of all the targeted patient-derived hiPSCs are shown in Figure 2F **,** with a mono-allelic insertion ranging from 40 to 80% and a bi-allelic insertion ranging from 6 to 40%, depending on the hiPSC line. Quantitative RNA expression analysis showed that *ARSB* mRNA expression after gene editing was 4-5-fold above the average expression in healthy controls (Figure 2G).

## 3.3 Differentiation of hiPSC to chondrogenic cells

**FIGURE 3:**
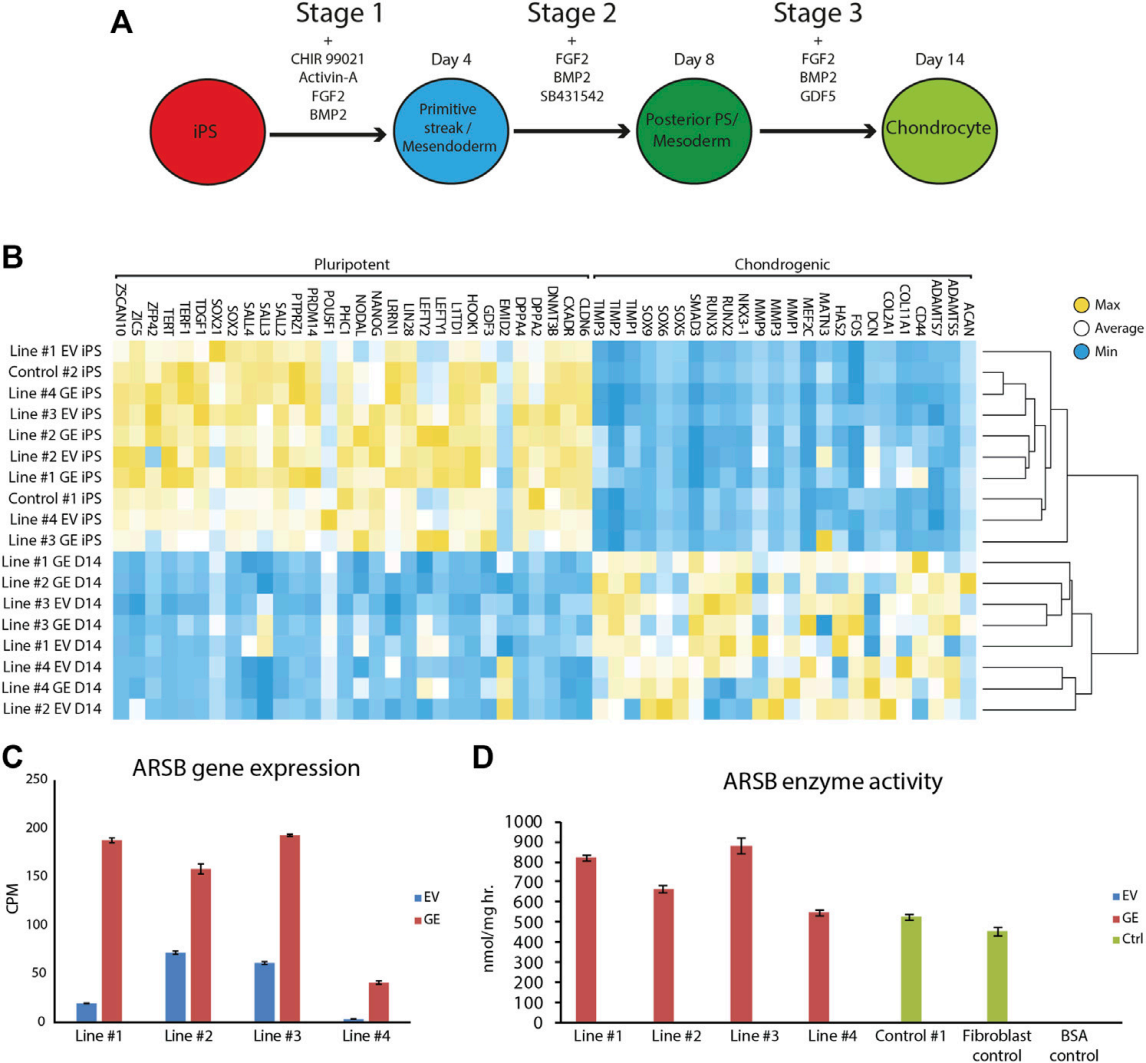
Differentiation of hiPSCs into chondrogenic cells. **(A)** Schematic representation of the refined 14-day differentiation protocol. Different factors are added to the media during the three stages. Cells are passaged at the end of stage 1 and 2 at day 4 and 8, respectively. **(B)** Expression analysis of pluripotent and chondrogenic genes show a loss of pluripotency and an increase in expression of chondrogenic genes after the chondrogenic differentiation. Expression of POU5F1 was very high in hiPSC Line #3 EV relative to all other hiPSC lines, resulting in a shift of the mean expression value for this gene used to generated the heat map. However, all hiPSC lines showed downregulation of POU5F1 upon differentiation. **(C)** ARSB gene expression remained high after chondrogenic differentiation (*n* = 3). **(D)** Biochemical analysis showed a rescue of ARSB enzyme activity in chondrogenic cells (n = 3). GE: Gene edited with *ARSB* cDNA, EV: Gene edited with empty vector control. Data are expressed as means ± SE.

Gene edited hiPSCs (GE and EV) were differentiated into chondrogenic cells using a novel 14-day differentiation protocol. This protocol uses a chemically defined culture media and matrix-coated substrates, and is based on the sequential activation of signaling pathways that operate during development of cartilage (Figure 3A, Supplementary Table S3). With this modified protocol we achieved successful chondrogenic differentiation in every hiPSC tested (data not shown). Genome-wide mRNA expression analysis was performed using RNA sequencing to compare expression of genes involve in pluripotency and chondrogenic differentiation between hiPSCs and chondrogenic cells. High expression of pluripotency genes was observed in hiPSC cells, while these genes were not expressed at day 14 of differentiation. Conversely, chondrogenic genes were not expressed at the hiPSC stage but were upregulated at day 14 of differentiation (Figure 3B). Key chondrogenic genes that were expressed after 14 days of differentiation included *COL2A1*, and *SOX9*. *ARSB* RNA expression in chondrogenic cells was 2 to 12-fold increased in the GE compared to the EV cells (Figure 3C). Immunostaining showed that SOX9 was expressed with nuclear localization, while thionin staining showed production of extracellular GAGs after differentiation, confirming chondrogenic differentiation (Supplementary Figure S1).

## 3.4 Chondrogenic cells from MPS VI patients accumulate PAS+ material

**FIGURE 4:**
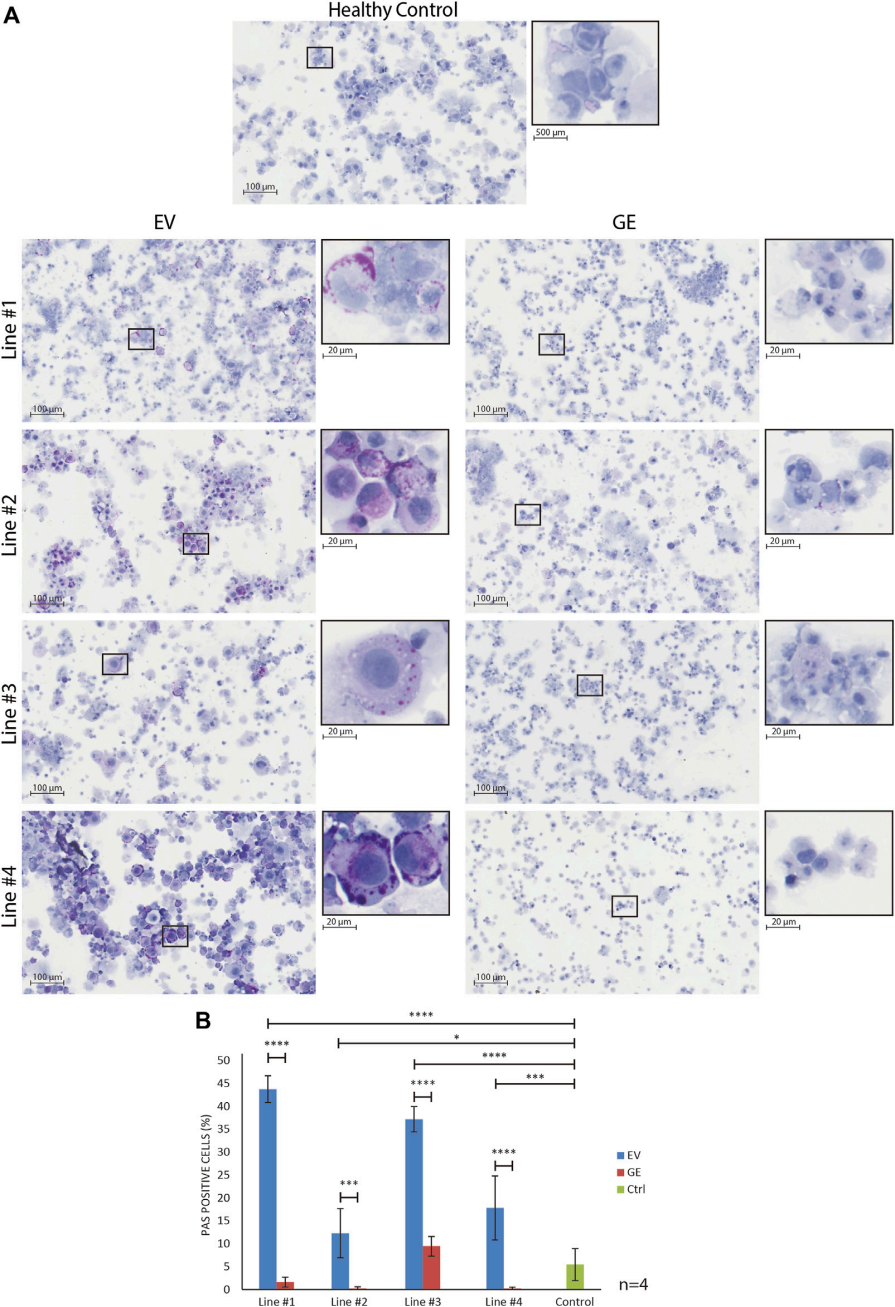
Normalization of inclusions after gene correction. **(A)** periodic acid–Schiff (PAS) staining of day 14 differentiated chondrogenic cells showed the accumulation of inclusions in EV cells, indicated by the increase of purple signal. The insert shows a higher magnification of selected cells to visualize the inclusions. **(B)** Quantification of PAS positive cells showed the normalization of inclusions after gene correction. Data represent means ± SD of 4 cytospins of independent cultures of chondrogenic cells. GE: Gene edited with *ARSB cDNA*, EV: Gene edited with empty vector control. Data are expressed as means ± SE. Statistical tests were performed with two-way ANOVA and Šídák multiple comparisons correction, *p ≤ 0.05, ***p ≤ 0.001, ****p ≤ 0.0001.

To assess whether hiPSC-derived chondrogenic cells accumulated carbohydrate macromolecules, we analyzed cytospins of GE and EV gene edited patient-derived cells using periodic acid–Schiff (PAS) staining (Figure 4). In EV cells, PAS-positive extranuclear areas were observed in the form of small to larger dots that in some cases seemed fused (see insets in Figure 4A. These PAS-positive areas were rare or absent in GE cells and in cells from a healthy control (Figure 4A). Quantification of the number of PAS positive cells confirmed this (Figure 4B). We conclude that hiPSC-derived chondrogenic cells generated from MPS VI patients store PAS-reactive material.

## 3.5 Genome-wide mRNA expression analysis of isogenic pairs

**FIGURE 5:**
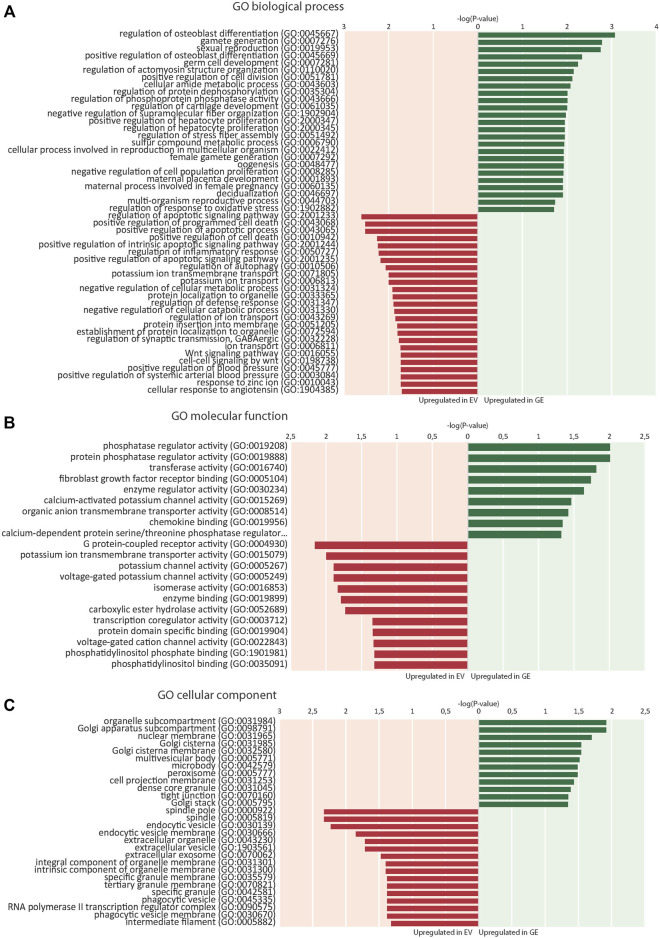
Enrichment of gene ontology (GO) terms. **(A)** 25 top dysregulated GO biological processes in EV and GE cells. **(B)** Significantly dysregulated GO molecular functions. **(C)** Significantly dysregulated GO cellular components. Upregulated processes in GE cells are indicated in green. Upregulated processes in EV cells are indicated in red. GE: Gene edited with *ARSB* cDNA, EV: Gene edited with empty vector control.

RNA sequence analysis was used in an unbiased approach to compare genome-wide changes in mRNA expression between chondrogenic cells from all 4 isogenic pairs. Biological triplicates clustered together (with one exception) and in general the differences between patients were larger than differences caused by gene correction, highlighting the need for isogenic controls (Supplementary Figure S3). This showed a total number of 703 differentially expressed genes when all 4 GE cells were compared to all 4 EV cells based on a fold change (FC) of >1.5, false discovery rate (FDR) of <0.05 and counts per million (CPM) > 0 (Supplementary Figure S4-S5). The subsets of upregulated genes in GE (420) and EV (283) were analyzed for enrichment of gene ontology (GO) terms using PANTHER (pantherdb.org). Analysis of biological processes revealed that 120 biological processes were upregulated in GE and 99 were upregulated in EV cells. The 25 most significant terms are shown for the upregulated genes of both sets in Figure 5A. Five over- or underrepresented biological processes were interesting in the light of cartilage and lysosomal homeostasis: cell growth and apoptosis, bone and cartilage development, Wnt signaling pathway, ion transport and regulation of metabolic processes. Some GO terms appeared high in the list, but upon inspection contained genes that had a function in cartilage (e.g. *NOG*, *BMP6* and *IGFBP5* in the category “osteoblast differentiation”) or had general roles (most genes in the category “germ cells/sexual reproduction”). Analysis of GO terms for molecular function showed that 9 groups were upregulated in GE and 12 that were upregulated in EV cells, mainly related to enzyme activity and ion channels (Figure 5B). Analysis of the GO terms for cellular component showed an upregulation of 12 components in GE cells, related to the Golgi apparatus and nuclear membrane, and 16 in EV cells, related to the spindle and vesicles (Figure 5C).

### 3.5.1 Bone and cartilage development

**FIGURE 6:**
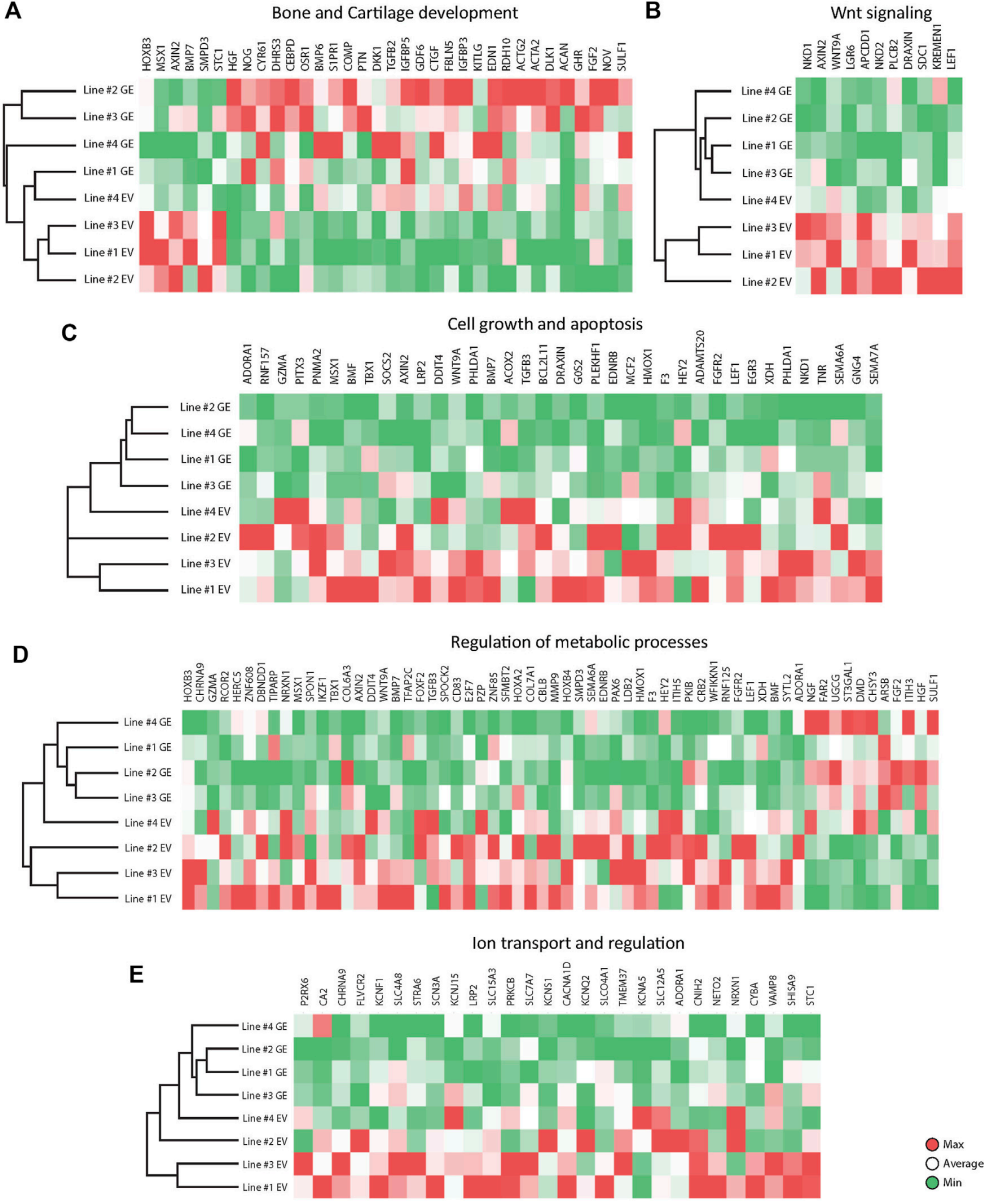
Expression analysis of differentially expressed genes between GE and EV. Expression analysis of differentially expressed genes involved in **(A)** Bone and Cartilage development, **(B)** WNT signaling, **(C)** Cell growth and apoptosis, **(D)** Metabolic processes, **(E)** Ion transport and regulation. GE: Gene edited with *ARSB* cDNA, EV: Gene edited with empty vector control.

**FIGURE 7:**
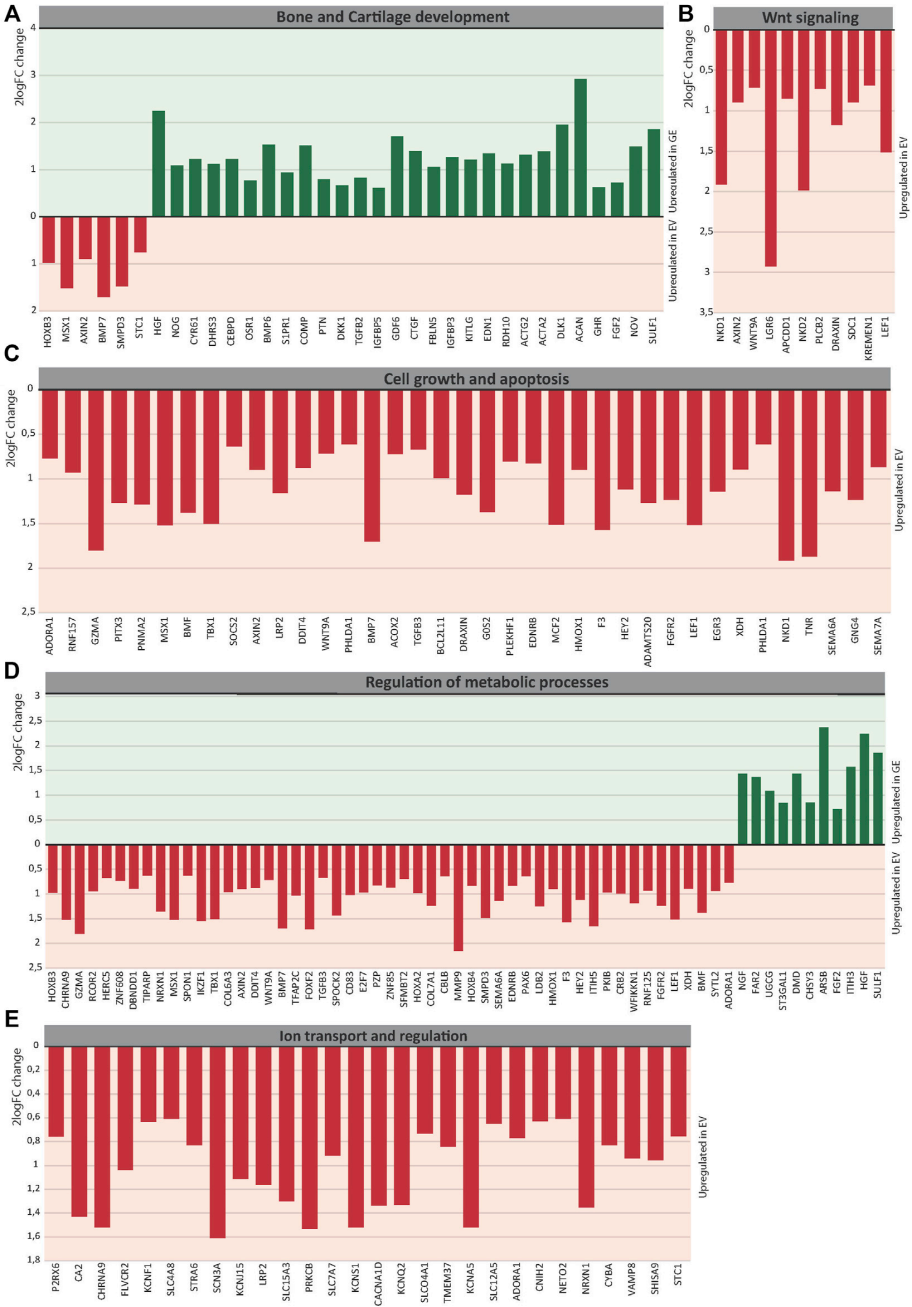
The 2^logFC^ of genes involved in **(A)** Bone and Cartilage development, **(B)** WNT signaling, **(C)** Cell growth and apoptosis, **(D)** Metabolic processes, **(E)** Ion transport and regulation. GE: Gene edited with *ARSB* cDNA, EV: Gene edited with empty vector control.

Several key genes involved in bone and cartilage development were differently expressed between GE and EV cells (Figure 6A): 28 genes were upregulated in GE and 6 in EV cells. Fold changes ranged from 1.5 for *IGFBP5* to 7,6 for *ACAN* (Figure 7A). Genes such as *TGFB2*, *BMP6*, *GDF6*, *PTN* and members of the CCN family *CYR61*, *NOV* and *CTGF* are all involved in the BMP/TGF-β signaling pathway and important in bone and cartilage development (Chang, 2016). *CTGF*, a BMP-7 inhibitor, was highly expressed in GE cells, and may explain the decreased expression of *BMP7* in EV cells. In addition, other proteins involved in extracellular matrix formation such as *ACAN*, *COMP* (Acharya et al., 2014; Coustry et al., 2018), *FBLN5* (Timpl et al., 2003; de Vega et al., 2009), and *SULF1* were upregulated in GE cells. These results suggest a dysregulated development of chondrogenic cells and extracellular matrix formation in MPS VI.

### 3.5.2 Wnt signaling pathway

GO analysis indicated a dysregulation of the canonical Wnt pathway as several signaling inhibitors such as *AXIN2* (Kikuchi, 1999; Jho et al., 2002; Katoh and Katoh, 2007; Larraguibel et al., 2015), *NKD1* (Katoh and Katoh, 2007; Angonin and Van Raay, 2013; Larraguibel et al., 2015), *NKD2* (Katoh and Katoh, 2007; Zhao et al., 2015), *APCDD1* (Shimomura et al., 2010; Cruciat and Niehrs, 2013), *DRAXIN* (Hutchins and Bronner, 2018), *KREMEN1* (Mao et al., 2002; Causeret et al., 2016), *WNT9A* (Ali et al., 2016) and *LEF1* (Steinke and Xue, 2014) were upregulated in EV (Figure 6B). Fold changes ranged from 1,6 for *KREMEN1* to 3,9 and 3.7 for *NKD2* and *NKD1*, respectively (Figure 7B). Apart from *WNT9A*, all other Wnt genes were either too lowly expressed or did not show a fold change above 1.5. *WNT2B* did show a >1.5-fold change upregulation in GE cells, but this was not significant with an FDR of 0.24 (data not shown). Upregulation of inhibitory genes may either indicate low Wnt levels and inactive β-catenin, or active Wnt signaling as inhibitors can be part of a feedback inhibition following pathway activation. Protein analysis showed an upregulation of active β-catenin in all three tested EV lines when compared to a healthy control (Supplementary Figure S6), which was normalized in line #4 after gene correction. These data suggest that the upregulation of Wnt inhibitors in MPS VI cells reflects a feedback inhibition mechanism following activation of the Wnt pathway.

### 3.5.3 Cell growth and apoptosis

GO analysis further indicated a dysregulation of cell growth and apoptosis in EV cells: 37 genes associated with negative regulation of cell growth and positive regulation of apoptosis were upregulated in EV cells (Figure 6C). Genes upregulated in EV cells that inhibit cell growth and promote apoptosis included: *MSX1* (Lallemand et al., 2009; Yue et al., 2018a; Yue et al., 2018b), *HMOX1* (Hill et al., 2005), *PHLDA1* (Zimnicka et al., 2017), *BMP7* (Piscione et al., 2001), *EGR3* (Zhang et al., 2017). SEMA6A (Shen et al., 2018), *BCL2L11* (Pinon et al., 2008; Koenig et al., 2014), *BMF* (Pinon et al., 2008), and *G0S2* (Welch et al., 2009). Fold changes ranged from 1.5 for *PHLDA1* to 3.7 for *NKD1* (Figure 7C). Protein analysis did not show the presence of cleaved Caspase 3 in any of the lines (Supplementary Figure S7), suggesting that dysregulation of gene expression did not lead to an induction of apoptosis at this stage.

### 3.5.4 Metabolic processes

The category metabolic processes contained 52 genes that were upregulated in EV cells and 11 that were upregulated in GE cells (Figure 6D). Fold changes ranged from 1.5 for *TIPARP* to 5.1 for *ARSB* itself (Figure 7D). Although most genes found in the analysis are factors indirectly involved in metabolic processes, several genes upregulated in EV cells are of particular interest. These include genes linked to the ECM such as *SPOCK2*, *MMP9*, *ITIH5* and the collagens *COL6A3* and *COL7A1*. Several genes related to GAG metabolism were upregulated in GE cells, including *SULF1*, *ITIH3*, *CHSY3*, involved in synthesis of CS, and *ST3GAL1*, involved in the synthesis of gangliosides including GM2 and GM3. Two E3 ubiquitin ligases, *RNF125* and *CBLB*, were upregulated in EV cells and are involved in proteasome-mediated protein degradation. Other genes upregulated in EV cells included *HEY2*, *SMPD3* and *XDH*. *HEY2* represses transcription by interaction with a histone deacetylase complex and is induced by the Notch signaling pathway (Weber et al., 2014). *SMPD3* catalyzes sphingomyelin hydrolysis. *XDH* catalyzes oxidative metabolism of purines. Other genes of interest upregulated in GE cells include *UGCG* and *FAR2*, involved in biosynthesis of glycosphingolipids and reduction of saturated fatty acids to fatty alcohols, respectively. These results indicate that ARSB deficiency may indirectly deregulate other metabolic processes besides GAG degradation.

### 3.5.5 Ion transport

GO analysis showed upregulation of 28 genes in EV cells that are involved in ion transport or regulation of ion transport (Figure 6E). Fold changes ranged from 1.5 for *SLC4A8* to 3 for *SCN3A* (Figure 7E). In addition, GO terms for molecular function analysis indicated the upregulation of potassium ion channel activity in EV cells (Figure 5B). Calcium-activated potassium channel activity was found to be upregulated in GE cells based on the molecular function analysis.

## 4 Discussion

In this study, we have generated a patient-derived *in vitro* disease model for MPS VI with isogenic controls, and used this to obtain insight in early stages of pathology in chondrogenic cells. We have applied this model to four patients and characterized cell biological and gene expression changes. This confirmed clinical information and previous reports using animal models, and provided novel insight in the early molecular changes in chondrocytes that are associated with MPS VI.

We used PAS to assess whether chondrocytes accumulated glycoprotein macromolecules. PAS positive vesicles were detected at day 14 of differentiation in disease chondrogenic cells relative to their isogenic controls. At present, the identity of the PAS positive material is unknown. We used a short protocol for PAS staining of cytospins that is unlikely capable of detecting charged GAGs such as DS and CS. Additional analysis using red oil staining ruled out that these were lipid droplets (Supplementary Figure S8). We hypothesize that they might represent secondary storage products, possibly GM2 or GM3, which are known to accumulate in MPS VI (Ballabio and Gieselmann, 2009). This would be compatible with the increased expression of *ST3GAL1* in MPS VI chondrocytes, as discussed below. Interestingly, the EM analysis of a cartilage biopsy from an MPS VI patient (see Figure 1) showed very heterogeneous shapes and forms of vesicles, suggesting that they might contain a mixture of storage products, which should be confirmed in future experiments. The fact that PAS positive material was already detected at day 14 of chondrogenic differentiation suggests that chondrogenic cells have an early onset pathology, which is in line with clinical information on hip abnormalities in MPS VI that likely develop before the age of 1 year (Oussoren et al., 2011; Oussoren et al., 2017).

Early disease onset in chondrogenic cells was also suggested by RNAseq analysis. Although gene expression of line #4 EV is closer to the transcriptional signature of GE cells than other EV lines, it was clearly distinct when compared to its isogenic gene edited counterpart. This highlights the power of using isogenic controls to correct for differences between individual cell lines. Key genes in bone and cartilage development were differentially expressed between EV and GE cells. Notably, genes involved in the TGF-β/BMP signaling, which plays a fundamental role in cartilage homeostasis, were downregulated in EV cells, including *BMP6* and *GDF6* (see also supplemental text). The TGF-β/BMP signaling pathway is known to be influenced by GAGs, but it is not fully understood how GAG accumulation in MPS influences its activity (Oussoren et al., 2011). Heparan sulfate containing proteoglycans (HSPG) can modulate BMPs and their antagonists (Khan et al., 2008) and chondroitin sulfate is linked to BMPs and TGF-β activity and intracellular localization (Kluppel et al., 2005; Alliston, 2010). TGF-β2 is one of the members of the TGF-β superfamily and was downregulated in EV cells. Downregulated members of the BMP family in EV cells included *BMP6* and *GDF6* (Chang, 2016; Xu et al., 2018; Tan Timur et al., 2019). *BMP6* stimulates differentiation of MSCs into chondrocytes and promotes the synthesis of chondrocytes (Rahman et al., 2015; Thielen et al., 2019; Ye et al., 2019). In addition, *BMP6* induces proteoglycan synthesis, which is particularly interesting in the context of the GAG accumulation in MPS VI (Bobacz et al., 2003; Chubinskaya et al., 2008; Thielen et al., 2019). *GDF6* is a member of the BMP family and a regulatory protein associated with the growth and differentiation of cartilaginous tissue (Wei et al., 2016). *PTN* is a TGF-β dependent heparin-binding growth-associated molecule and downregulated in EV cells, it is involved in a variety of processes in bone formation and stimulates chondrocyte proliferation (Tare et al., 2002; Pufe et al., 2007). The CCN family members *CYR61*, *NOV* and *CTGF* were upregulated in GE cells and are known to induce the expression of chondrogenic markers and to regulate TGF-β and BMP (Abreu et al., 2002; Holbourn et al., 2008; Nguyen et al., 2008; Maeda et al., 2009). *CYR61* is a direct target of canonical Wnt/beta-catenin signaling and is involved in osteogenic differentiation and bone healing (Si et al., 2006). *NOV* is involved in the maintenance of articular cartilage and may inhibit osteoarthritis (Janune et al., 2017; Huang et al., 2019). *CTGF* is involved in the regulation of chondrogenesis and is a BMP-7 inhibitor, possibly mediating the downregulation of *BMP7* in GE cells (Abreu et al., 2002; Nguyen et al., 2008; Maeda et al., 2009). *COMP* was downregulated in EV and encodes an extracellular matrix protein which plays an important role in cartilage matrix organization through interaction with other extracellular matrix proteins (Acharya et al., 2014; Coustry et al., 2018; Maly et al., 2021). Disease-associated variants in *COMP* cause impaired cartilage development leading to skeletal abnormalities such as short stature and skeletal dysplasias (Briggs et al., 1995; Hecht et al., 1995; McKeand et al., 1996; Hecht et al., 1998; Unger and Hecht, 2001; Hecht et al., 2005; Posey and Hecht, 2008). These results suggest that the accumulation of intralysosomal GAGs in MPS VI impairs early chondrogenic development by interfering with TGF-β/BMP signaling.

Several Wnt inhibitors of the Wnt/β-catenin signaling pathway were upregulated in EV cells including *AXIN2*, *NKD1*, *NKD2*, *APCDD1*, *DRAXIN*, *KREMEN1*, *WNT9A* and *LEF1*. *AXIN2* is a scaffold protein of the β-catenin destruction complex and promotes phosphorylation of β-catenin and its consequent degradation. *NKD1* and *NKD2* interact with AXIN2 to inhibit β-catenin in the canonical Wnt signaling pathway (Miller et al., 2009). *APCDD1* can co-precipitate with Wnt3A and LRP5 and likely inhibits Wnt/β-catenin signaling by preventing formation of the Wnt receptor complex (Shimomura et al., 2010). The zebrafish homologue of *DRAXIN*, *Neucrin*, has been suggested to inhibit the stabilization of β-catenin during canonical Wnt signalling (Miyake et al., 2009). *KREMEN1* is a transmembrane protein that acts as a receptor for Dickkopf-1 with which it functionally cooperates to inhibit the Wnt/β-catenin signaling. *WNT9A* has been suggested as a non-canonical ligand to inhibit β-catenin (Ali et al., 2016). *LEF1* is a key target in the Wnt signaling pathway and can negatively regulate the expression of Wnt signaling genes by binding to Groucho-related corepressors (Steinke and Xue, 2014). This upregulation of Wnt inhibitors and the upregulation of active β-catenin on protein level, suggests that the Wnt signaling pathway is dysregulated in chondrogenic cells with MPS VI. Interestingly, a link between GAGs and Wnt signaling has been made previously: biglycan, a protein core with two CS or DS GAG chains, affects the Wnt signaling pathway through a direct interaction of the core protein with the LRP6 receptor and its Wnt ligand (Berendsen et al., 2011). The Wnt signaling pathway plays a crucial role in bone and cartilage development (Narcisi et al., 2015; Kobayashi et al., 2016; Usami et al., 2016), and downregulation of Wnt2 and beta-catenin can inhibit cell proliferation and induce apoptosis (You et al., 2004; Pu et al., 2009; Zimmerman et al., 2013), suggesting that chondrogenic cells with MPS VI undergo impaired chondrogenic development and reduced cell proliferation via intereference with Wnt signalling.

Evidence for increased apoptotic stimuli and decreased cell growth in MPS VI was obtained by upregulation of 37 genes associated with these processes in EV cells. These included *MSX1*, *HMOX1*, *PHLDA1*, *BMP7*, *EGR3*, *SEMA6A*, *BCL2L11*, *BMF* and *G0S2*. *MSX1* has been shown to inhibit cell growth and induce apoptosis by inhibiting the Notch signaling pathway and maintaining cyclin D1 expression (Hu et al., 2001; Lallemand et al., 2009; Yue et al., 2018a; Yue et al., 2018b). Upregulation of *PHLDA1* expression leads to reduced cell growth and increased apoptosis (Neef et al., 2002; Nagai, 2016; Zimnicka et al., 2017). *BMP7* contributes to cell cycle arrest at the G1/S checkpoint and stimulates apoptosis via Smad1-dependent and Smad1-independent pathways (Piscione et al., 2001; Klose et al., 2011). SEMA6A induces apoptosis via the cytosolic region of the SEMA domain through association with Fas-associated protein with death domain (FADD) (Shen et al., 2018). *EGR3* inhibits cell proliferation and induces apoptosis via upregulation of Fas ligand (Zhang et al., 2017)*. BCL2L11*, also known as *BIM*, *BMF* and *G0S2* interact together to promote apoptosis (Ramjaun et al., 2007). Increased apoptotic stimuli in MPS VI chondrocytes is likely due to the GAG accumulation, which is consistent with findings from Simonaro et al. (Simonaro et al., 2008) who showed that GAG accumulation leads to apoptosis of articular chondrocytes. However, in this case chondrocyte apoptosis was due to the activation of the TLR-4 signaling pathway followed by the release of proinflammatory cytokines. In contrast, we showed increased apoptotic stimuli in the absence of proinflammatory cytokines, suggesting the existence of an intrinsic non-inflammatory mechanism.

Interestingly, several genes linked to GAG metabolism were dysregulated in EV cells. *SPOCK2*, *MMP9* and *ITIH5* were upregulated and *SULF1*, *ITIH3*, *CHSY3* and *ST3GAL1* were downregulated compared to GE cells. *SPOCK2* encodes the protein Testican-2, a proteoglycan that binds to glycosaminoglycans and forms part of the extracellular matrix. *MMP9* encodes an enzyme belonging to the matrix metalloproteinase family with substrates including aggrecan and collagen and non-ECM substrates such TGF-β1 (Ma et al., 2015). *ITIH5* is involved in extracellular matrix stabilization. *SULF1* encodes a heparan sulfate endosulfatase which removes sulfate groups from chains of heparan sulfate proteoglycans. *ITIH3* encodes a subunit of the pre-alpha-trypsin inhibitor complex which binds to bind hyaluronic acid and stabilizes the extracellular matrix. *CHSY3*, also known as CSS3 or chondroitin sulfate synthase, is a glycosyltransferase that is involved in transferring GlcUA and GalNAc to the nonreducing terminus of chondroitin sulfate. Its downregulation in MPS VI may indicate a feedback mechanism induced by CS accumulation to reduce the synthesis of CS. *ST3GAL1* encodes a sialyltransferase that catalyzes the transfer of sialic acid from cytidine monophosphate-sialic acid to galactose-containing substrates such as GAGs (Wu et al., 2018). It is also involved in the synthesis of GM2 and GM3, known secondary storage products in many lysosomal storage disorders including MPS VI (Ballabio and Gieselmann, 2009). These findings indicate that MPS VI chondrogenic cells have disturbed metabolism of GAGs and other ECM components, including feedback inhibition of CS synthesis and disturbed ganglioside metabolism.

Genes involved in ion transport of calcium and potassium and encoding other ion channels were upregulated in EV cells. Genes involved in potassium transport included *KCNF1*, *KCNJ15*, *KCNS1*, *KCNA5*, *KCNQ2* and *SLC12A5*. Genes linked to calcium transport include *FLVCR2*, *CACNA1D*, *TMEM37* and *STC1*. Other genes upregulated in EV cells included *CHRNA9*, *SCN3A*, and *P2RX6*, a ligand-gated ionic channel, a voltage-gated sodium channels and ATP-gated ion, respectively. Changes in ion channels have been linked previously to osteoarthritic cartilage (Lewis and Barrett-Jolley, 2015; Bertram et al., 2016). In addition, extracellular GAG deposits in tissue causes increased absorption of water which results in inflated tissue (Hampe et al., 2020). The intralysosomal accumulation of GAGs in EV cells might cause osmotic stress as GAGs can retain water up to 100 times up to its weight. The dysregulation of ion channels might be a regulatory mechanism to compensate for the difference in cell osmolarity (Erickson et al., 2001; Erickson et al., 2003; Kurita et al., 2015). In addition, accumulation of GAGs is suggested to compromise the integrity of lysosomal membranes (Hampe et al., 2020), which can lead to a dysregulation of ion content in the lysosome.

In summary, we have generated a disease model for the cartilage pathology in MPS VI based on hiPSCs and isogenic controls using CRISPR/Cas9. This has proven to be a powerful approach to detect the early cellular and molecular changes in chondrogenic cells that ultimately lead to the severe cartilage pathology in MPS VI patients. As the approach is generic, it should be generally applicable to model genetic disorders that affect cartilage. Future work should focus on further development of the model into actual cartilage that is subject to mechanical loading that mimics natural loading of weight bearing joints that occurs in human individuals.

## Data Availability

The data discussed in this publication have been deposited in NCBI’s Gene Expression Omnibus and are accessible through GEO Series accession number GSE218101 (https://www.ncbi.nlm.nih.gov/geo/query/acc.cgi?acc=GSE218101).
