# Supplementary material for: Modeling cartilage pathology in mucopolysaccharidosis VI using iPSCs reveals early dysregulation of chondrogenic and metabolic gene expression
Source: Front Bioeng Biotechnol. 2022 Dec 6;10:949063. doi: 10.3389/fbioe.2022.949063 (PMC9763729; doi:10.3389/fbioe.2022.949063)
Supplement: Supplementary file 1 [file DataSheet1.pdf]

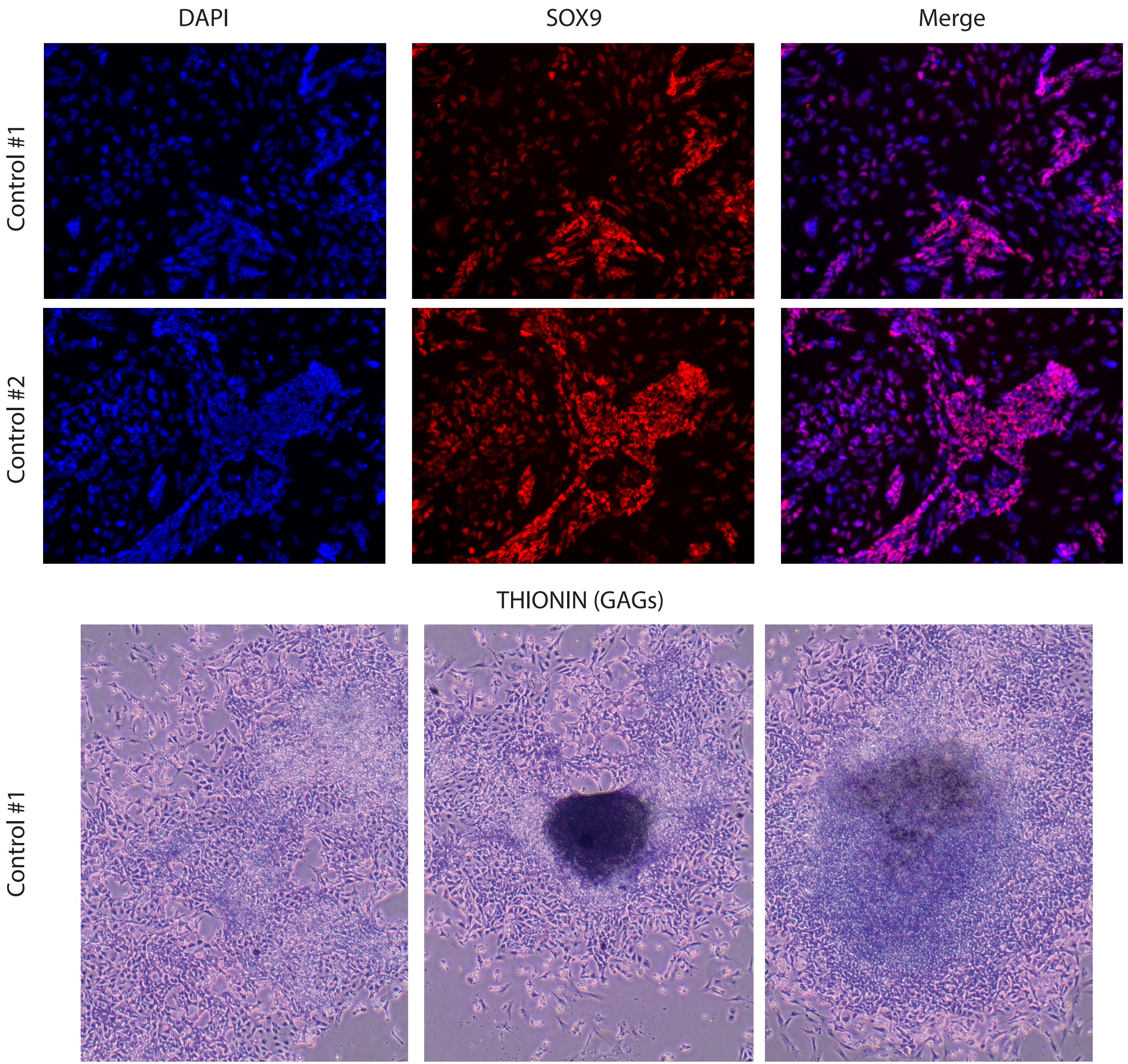

Figure S1. Immunofluorescence of SOX9 in differentiated chondrogenic cells of two control lines and thionin staining of Control #1 in triplicate. SOX9 immunostaining was performed as described in Oldershaw et al. (2010) and thionin staining was performed as described in Cleary et al. (2016)

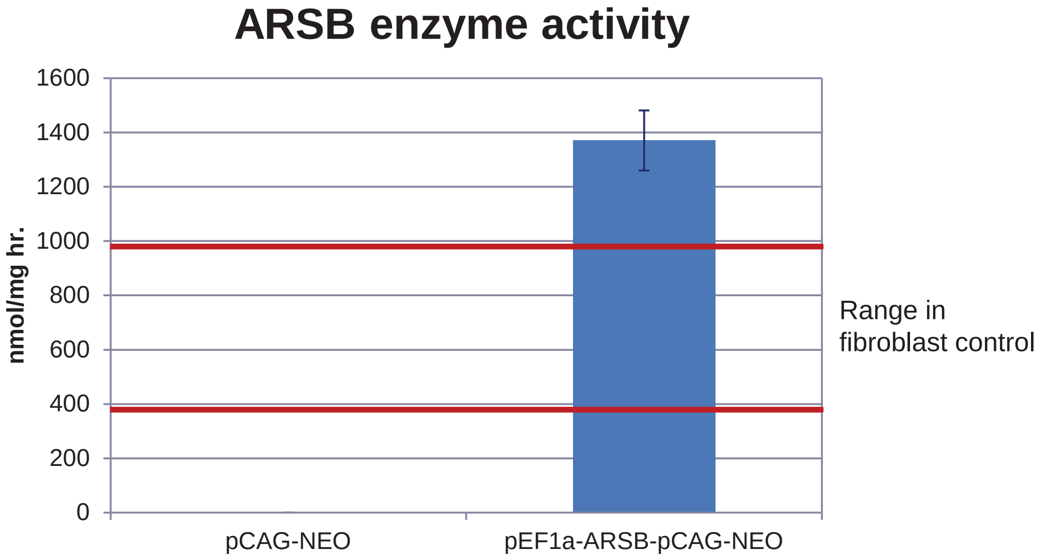

Figure S2. ARSB enzyme activity in HeLa Tk- cells following transfection of pEF1a-ARSB-pCAG-NEO. ARSB enzyme activity was measured in HeLa Tk- cells after selection with G418. No background activity was measured after transfection with pCAG-Neo as a negative control. The red solid lines indicate the ARSB enzyme activity levels in the human skin fibroblast sample used for this assay.

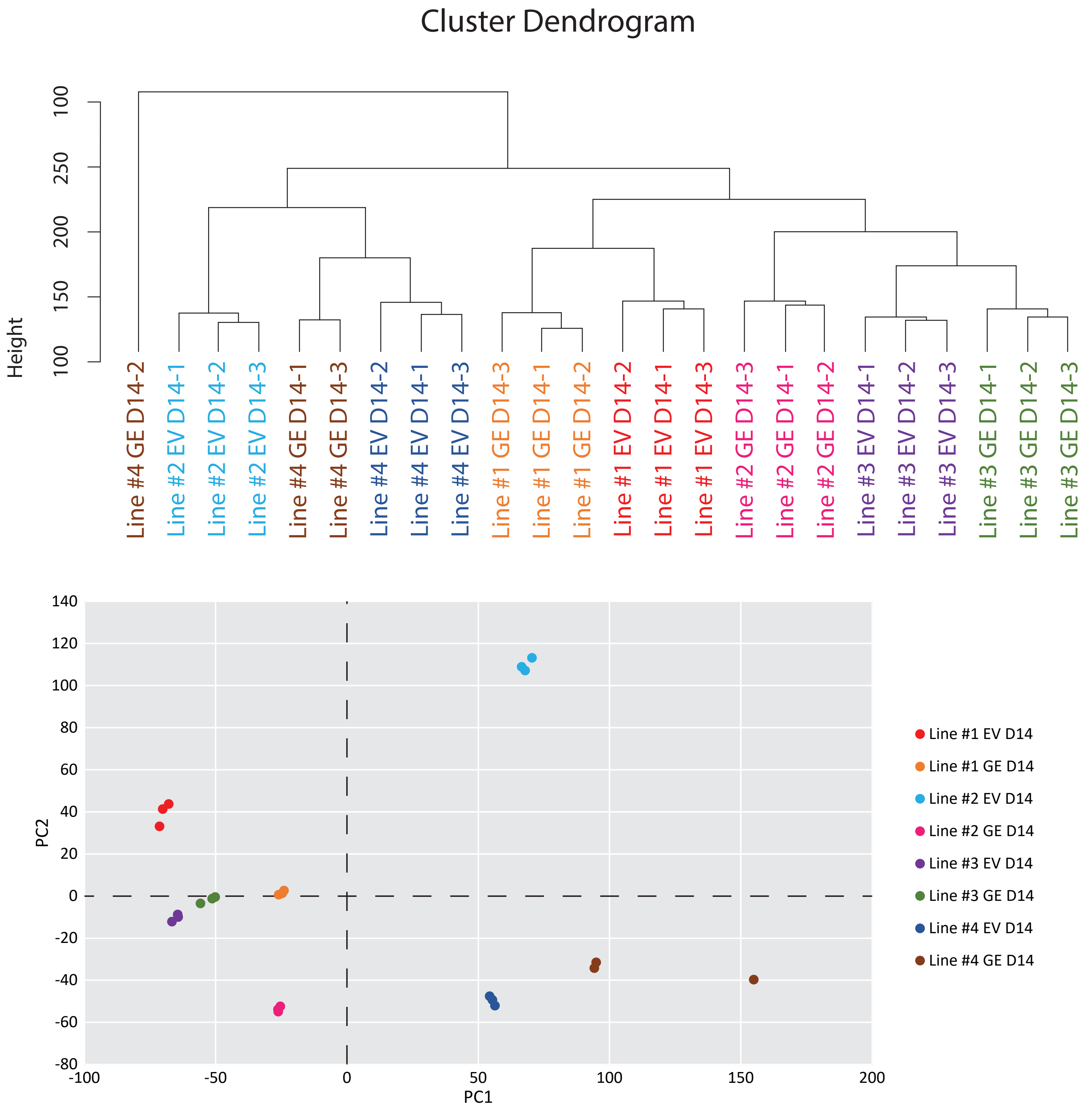

Figure S3. Cluster analysis and principal component analysis of all lines. Colors represent the triplicate for each line, GE: Gene edited, EV: Empty vector control.

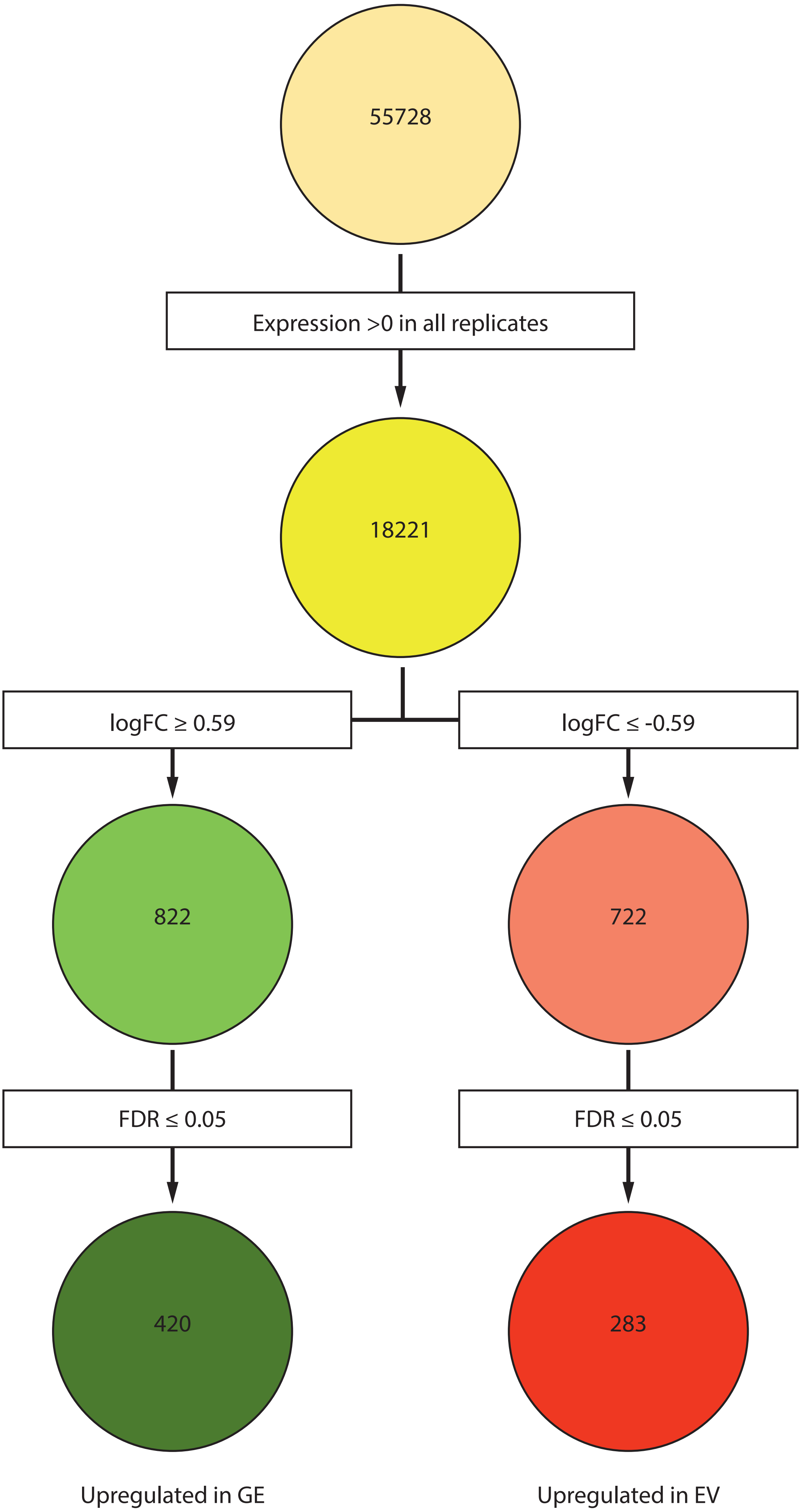

Figure S4. Flowchart of data analysis. Genes with a CPM of 0 in one or more samples were filtered out. Selection of genes with a 2logFC of >0.59 and an FDR of <0.05 resulted in the identification of 420 genes that were upregulated in GE cells. Selection of genes with a 2logFC of <-0.59 and an FDR of <0.05 resulted in the identification of 283 upregulated genes in empty vector cells. GE: Gene edited with ARSB cDNA, EV: Gene edited with empty vector control.

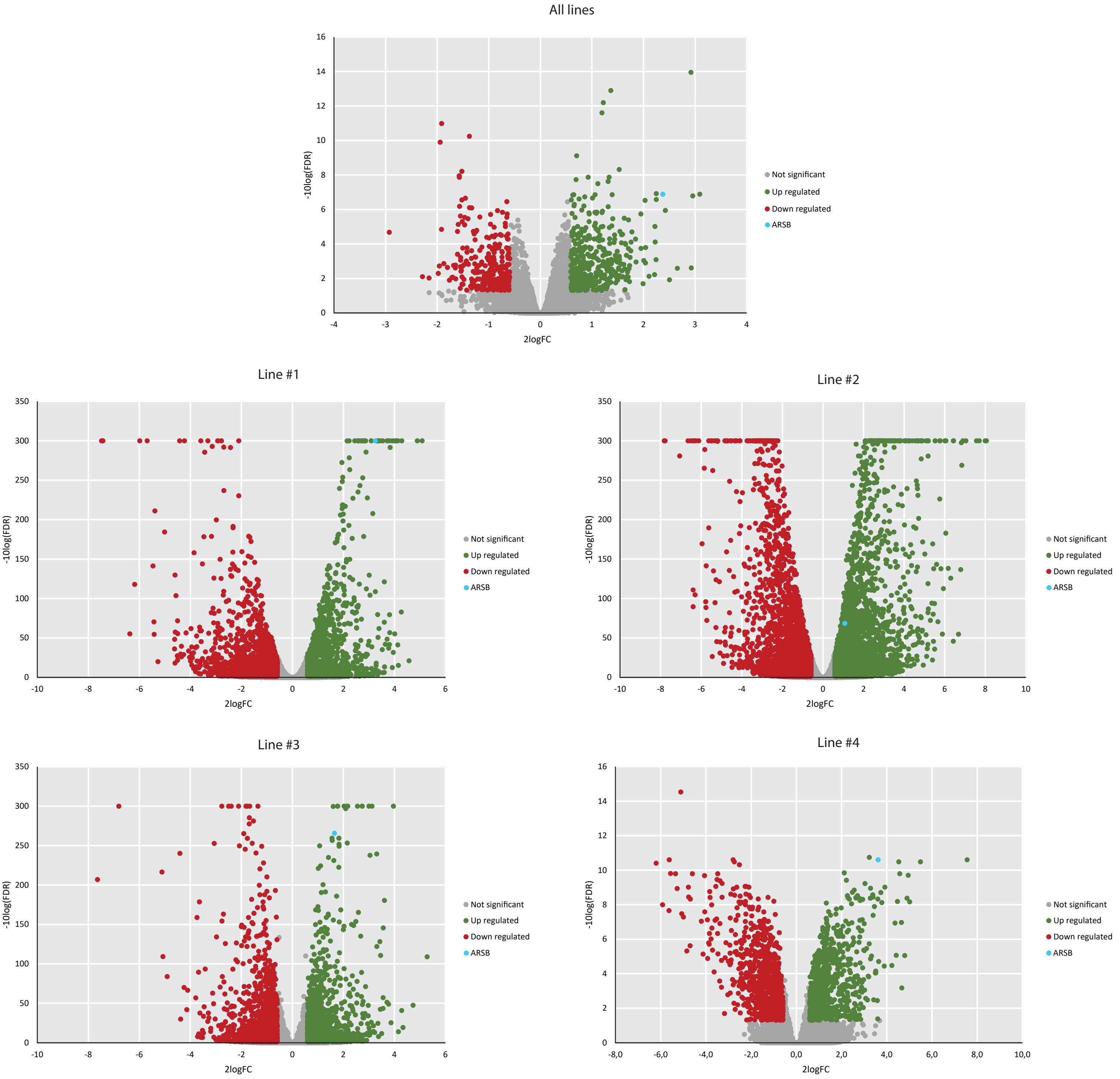

Figure S5. Vulcano plots of all samples together and individual isogenic pairs. Each dot represents a single gene, those in red have a 2^{logFC} of <-0.59 and an FDR of <0.05, those in green have a 2^{logFC} of >0.59 and an FDR of <0.05. ARSB is highlighted in blue. Not significantly changed genes are displayed in gray.

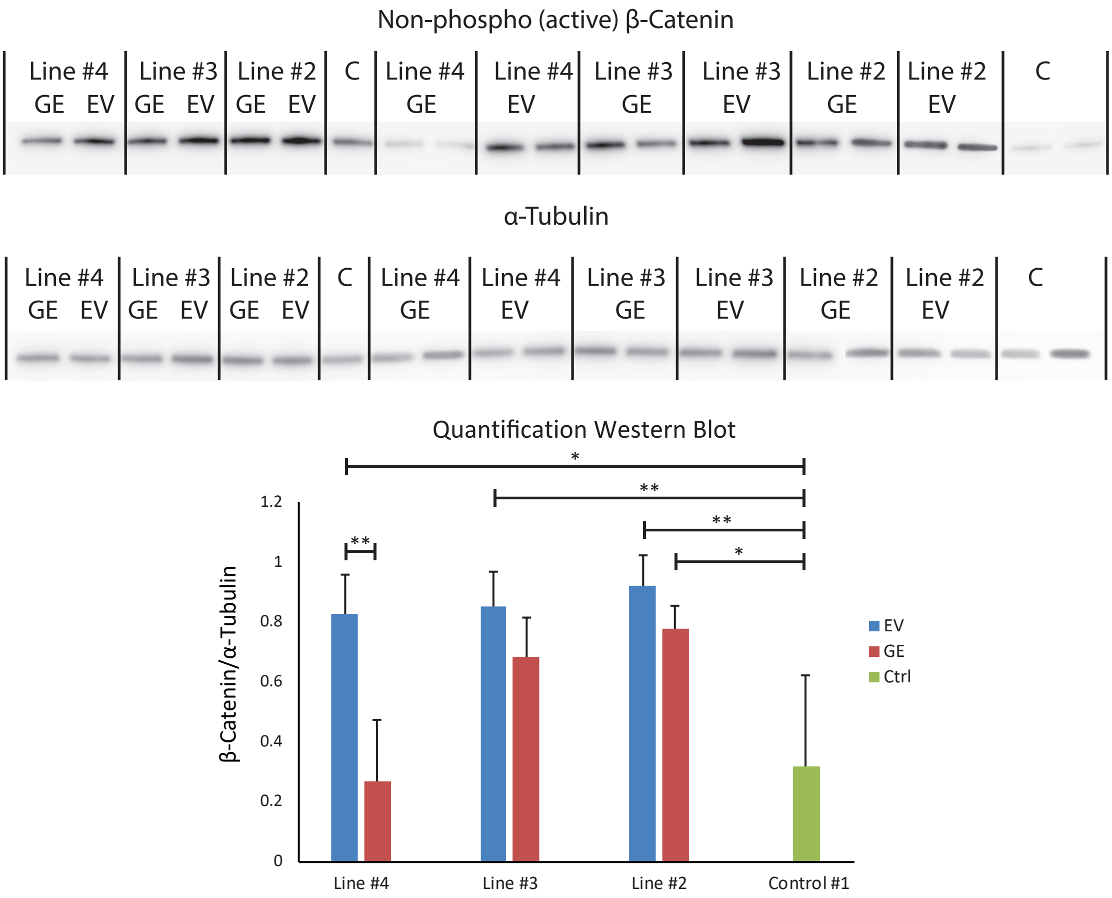

Figure S6. Western blot analysis of β-Catenin and α-Tubulin with quantification. GE: Gene edited with ARSB cDNA, EV: Gene edited with empty vector control, C: Control #1. Data are expressed as means ± SE. Statistical tests were performed with two-way ANOVA and Šídák multiple comparisons correction, *$p \leq 0.05$, **$p \leq 0.01$.

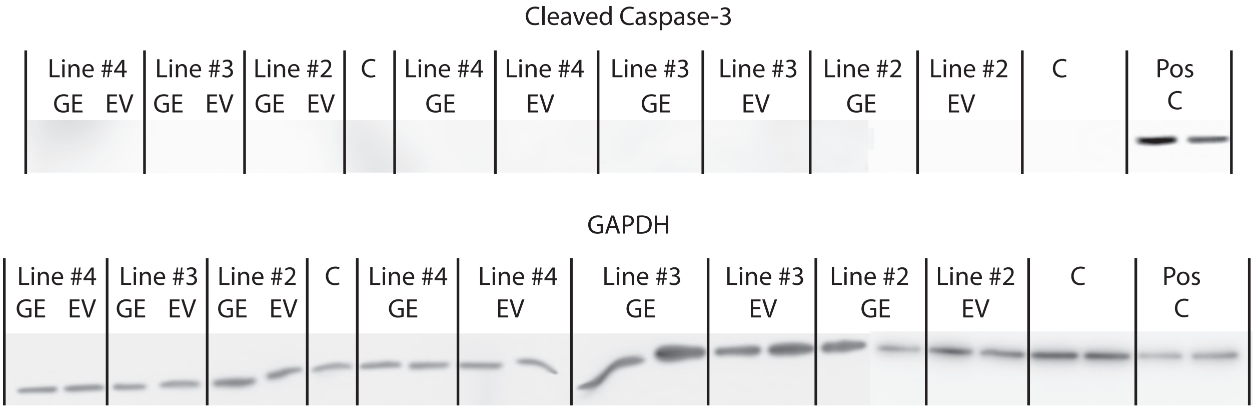

Figure S7. Western blot analysis of Cleaved Caspase 3 and GAPDH with quantification. GE: Gene edited with ARSB cDNA, EV: Gene edited with empty vector control, C: Control #1, Pos C: Positive control, Control #1 cells incubated 1 h at 42°C to induce apoptosis.

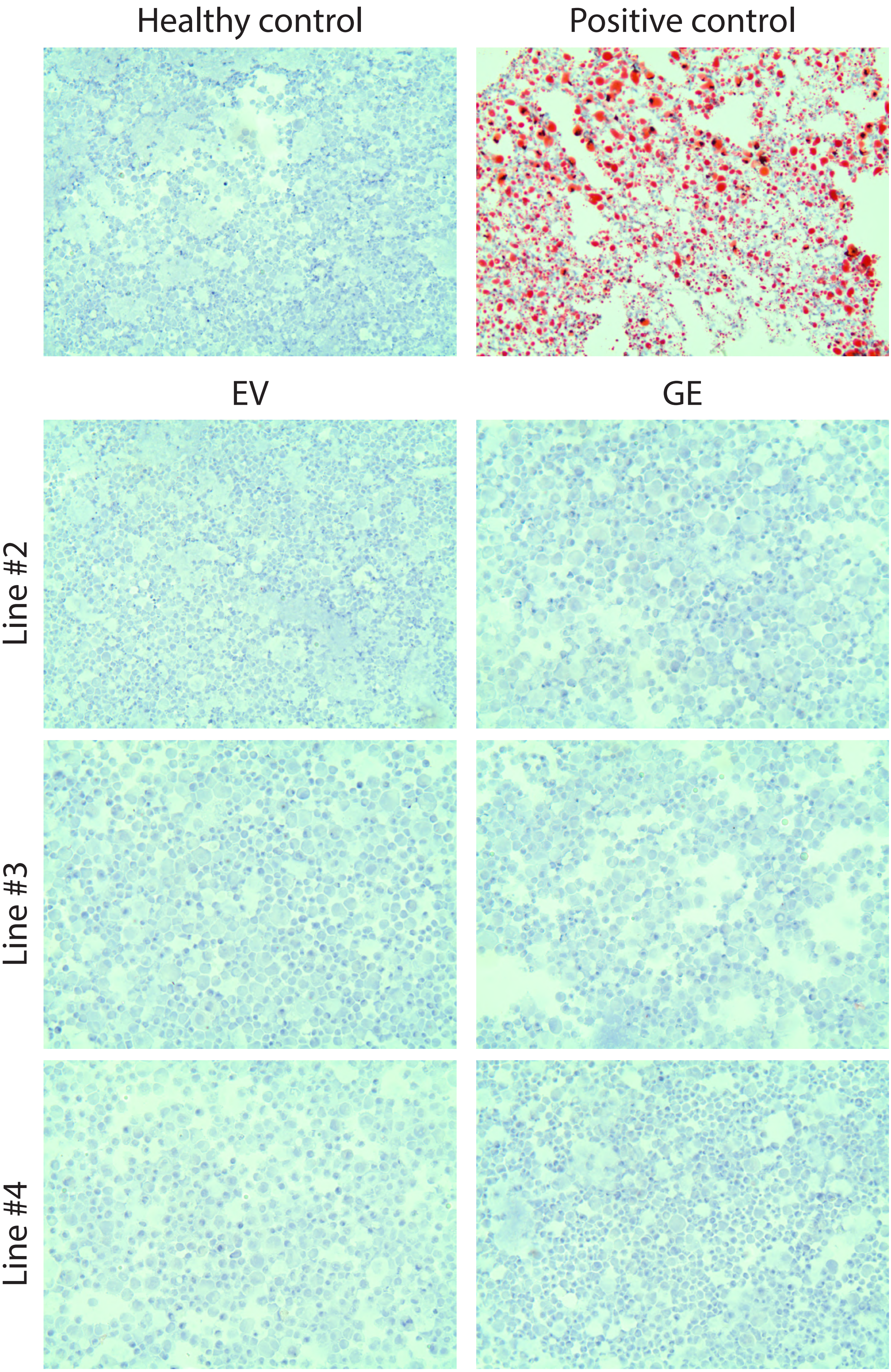

Figure S8. Red oil staining of differentiated chondrogenic cells on cytospins. GE: Gene edited with ARSB cDNA, EV: Gene edited with empty vector control. The positive control consists of a diagnostic adipose tissue section.

Table S1. Off target analysis for AAVS1 gRNA using CRISPRscan

Site type				Gg18NGG
gRNA				TCACCAATCCTGTCCCTAG
Off-targets (top 30 shown out of 657)				
	All	Seed	CFD	
19:55115744[....................]+	✓	✓	0.74	AAVS1 target site
2:178603021[.....XX............X]-	-	-	0.52	
14:97329501[.X...X.....XX...X...]+	-	-	0.38	
2:116084549[.....X..........X..X]-	-	-	0.34	
8:71621720[.X......X.XXX.......]-	-	-	0.28	
1:182304836[X.X..X......X.......]+	-	-	0.27	
9:86900721[X.......XX......X...]+	-	-	0.24	
15:36995651[X.XX..X.............]+	-	-	0.24	
16:55430763[.....X............X.]+	-	-	0.24	
1:12523844[XX...X...........X..]+	-	-	0.24	
17:20511580[XX.....XX...........]+	-	-	0.24	
17:16839915[XX.....XX...........]+	-	-	0.24	
17:18432804[XX.....XX...........]-	-	-	0.24	
7:146900872[X...XX.X............]+	-	-	0.23	
11:2211239[X....X.....X.......X]+	-	-	0.21	
22:48160907[.X...X......X......X]+	-	-	0.2	
X:14923806[X....X....X........X]-	-	-	0.2	
3:69406638[.X..X...X.X.....X...]+	-	-	0.19	
4:24089264[........XX.........X]-	-	-	0.19	
9:109440579[.X..X.....X......X.X]+	-	-	0.18	
5:78913049[X......X....X......X]+	-	-	0.16	
14:76966187[....XX......X.......]+	-	-	0.16	
11:83702980[X...X......X.....X..]+	-	-	0.16	
2:106823886[.X...X..........X.XX]+	-	-	0.15	
5:133240963[.XX....X.X.....X....]+	-	-	0.15	
8:66370990[X...XX..............]-	-	-	0.14	
10:70362938[.....X..........X.X.]-	-	-	0.14	
1:2859981[.X......X.X..X.X....]-	-	-	0.13	
9:129714498[.X.....X.........X.X]+	-	-	0.12	
10:130964023[.X.....XX...X....X..]-	-	-	0.12	

Table S2. Primers used for cDNA cloning of ARSB and genotyping gene edited hiPSCs.

**Name**	**Sequence 5'-3'**	**Company**
cDNA cloning of ARSB		
Fw_cDNA_ARSB	GGTGGTGAATTCGGTGGTTTAATTAATAAAACGACGGCCAGTGAATTG	IDTDNA
Rv_cDNA_ARSB	GGTGGTGCGGCCGCGGTGGTATGCATTCTACATCCAAGGGCCCCA	IDTDNA
Genotyping iPS clones		
Set 1 forward	TTCCCAGGGCCGGTTAATGT	IDTDNA
Set 1 reverse	GCTCTGGGCGGAGGAATATG	IDTDNA
Set 2 forward	CCTGAGTCCGGACCACTTTG	IDTDNA
Set 2 reverse	CACCGGTTCAATTGCCGAC	IDTDNA

Table S3. Chondrogenic differentiation protocol for stages 1–3. Revitacell: RevitaCell™ Supplement (100X); VN: Vitronectin XF; VN:Gel0,1%: Vitronectin XF/Gelatin 0,1%

Day	CHIR 99021	Activin-A	FGF2	BMP2	SB431542	GDF5	Revitacell	Media	Coating	Split
0	Plate out 500k to 1M iPS cells									
1	2 µM	50 ng/mL						2 mL	VN	
2	2 µM	25 ng/mL	40 ng/mL					2 mL	VN	
3	2 µM	10 ng/mL	40 ng/mL	5 ng/mL				2 mL	VN	
4			40 ng/mL	5 ng/mL				2 mL	VN	
5			40 ng/mL	5 ng/mL	1 µM		10 µl/mL	2 mL	VN	1/5 to 1/8
6			40 ng/mL	5 ng/mL	1 µM			2 mL	VN	
7			40 ng/mL	5 ng/mL	1 µM			2 mL	VN	
8			40 ng/mL	5 ng/mL	1 µM		10 µl/mL	2 mL	VN:Gel0,1%	1/4 to 1/6
9			40 ng/mL	5 ng/mL		20 ng/mL		2 mL	VN:Gel0,1%	
10			40 ng/mL	5 ng/mL		20 ng/mL		2 mL	VN:Gel0,1%	
11			40 ng/mL			40 ng/mL		2 mL	VN:Gel0,1%	
12			40 ng/mL			40 ng/mL		2 mL	VN:Gel0,1%	
13			40 ng/mL			40 ng/mL		2 mL	VN:Gel0,1%	
14	Termination									

We adapted the chondrogenic differentiation protocol for hiPSCs based on our previous protocol (Oldershaw et al., 2010) to increase its robustness. The highly unstable Wnt3a was replaced by the small molecule CHIR-99021, a selective GSK-3α/β inhibitor and a Wnt/β-catenin signaling pathway activator. For activin inhibition in stage two of the protocol, Follistatin was replaced by the small molecule SB431542, which is a more selective activin inhibitor (Chen et al., 2012). BMP4 was replaced with BMP2, which has previously been shown to be more potent at driving chondrogenic differentiation (Wang et al., 2019)
